# Issues experienced while administering care to patients with dementia in acute care hospitals: A study based on focus group interviews

**DOI:** 10.3402/qhw.v10.25828

**Published:** 2015-02-24

**Authors:** Risa Fukuda, Yasuko Shimizu, Natsuko Seto

**Affiliations:** 1 Nursing and Health Science, Graduate School of Medicine, Ehime University, Ehime, Japan; 2 Division of Health Sciences, Graduate School of Medicine, Osaka University, Osaka, Japan

**Keywords:** Difficulties in administering care, elderly, focus groups, geriatric nursing, KJ method, patient care management

## Abstract

**Objective:**

Dementia is a major public health problem. More and more patients with dementia are being admitted to acute care hospitals for treatment of comorbidities. Issues associated with care of patients with dementia in acute care hospitals have not been adequately clarified. This study aimed to explore the challenges nurses face in providing care to patients with dementia in acute care hospitals in Japan.

**Methods:**

This was a qualitative study using focus group interviews (FGIs). The setting was six acute hospitals with surgical and medical wards in the western region of Japan. Participants were nurses in surgical and internal medicine wards, excluding intensive care units. Nurses with less than 3 years working experience, those without experience in dementia patient care in their currently assigned ward, and head nurses were excluded from participation. FGIs were used to collect data from February to December 2008. Interviews were scheduled for 1–1.5 h. The qualitative synthesis method was used for data analysis.

**Results:**

In total, 50 nurses with an average experience of 9.8 years participated. Eight focus groups were formed. Issues in administering care to patients with dementia at acute care hospitals were divided into seven groups. Three of these groups, that is, problematic patient behaviors, recurrent problem, and problems affecting many people equally, interact to result in a burdensome cycle. This cycle is exacerbated by lack of nursing experience and lack of organization in hospitals. In coping with this cycle, the nurses develop protection plans for themselves and for the hospital.

**Conclusions:**

The two main issues experienced by nurses while administering care to patients with dementia in acute care hospitals were as follows: (a) the various problems and difficulties faced by nurses were interactive and caused a burdensome cycle, and (b) nurses do their best to adapt to these conditions despite feeling conflicted.

The number of patients with dementia worldwide was estimated as 24 million in 2001, with the number expected to double every 20 years (Qiu, De Ronchi, & Fratiglioni, 2007). The number of patients with dementia in Japan has also grown continually: in 2005, the prevalence of dementia among older adults aged >65 years was 7.6% (approximately 1.89 million people), with the number expected to reach 8.9% (2.92 million people) through population aging by 2020 (Ministry of Health, Labour and Welfare, 2005). Admission of patients with dementia to acute care hospitals for treatment of acute diseases has also increased (Mukadam & Sampson, 2011; Sandberg, Gustafson, Brannstrom, & Bucht, 1998) in Japan (Ministry of Health, Labour and Welfare, 2005) because of this trend. Symptoms of dementia are aggravated in the acute care environment (Cunningham & Archibald, 2006; Fetzer, 1999; King & Watt, 1995; Martin & Haynes, 2000), but nurses in acute care hospitals are expected to minimize the aggravation of dementia through provision of adequate care.

However, nurses in acute care hospitals may be unable to provide care adequate to meet the needs of patients with dementia. Many patients with dementia are admitted to long-term care facilities or psychiatric wards (Awata & Watari, 2007; Lithgow, Jackson, & Browne, 2011; Miura et al., 2005; Sandberg et al., 1998; Yamasaki & Kodama, 1995). Particularly in Japan, until about 2008, measures of dementia care placed importance on aspects of nursing support, such as basic maintenance of nursing care services and construction of the community care system (Awata, 2010). As a result, 65.2% of dementia inpatients were in psychiatric wards, and 24.9% were in recuperation facilities (Miura et al., 2005). Therefore, nurses in Japanese acute care hospitals have had few opportunities to care for patients with dementia until recently. Thus, they may face various problems and difficulties in caring for patients with dementia.

Previous studies have investigated the difficulties and experiences of nurses involved in the care of patients with dementia in acute care hospitals. Eriksson and Saveman (2002) described ethically difficult situations that can lead to abuse, difficulties related to disorderly conduct among patients with dementia, and problems related to the organization of acute care as an obstacle to good nursing care for patients with dementia. With regard to experiences in an acute care ward at a university hospital, a different study revealed several issues that nurses face in relation to dementia care, including responsibility for patients, frustrations with regard to time, frustrations with regard to lack of organization, divided tasks, and working alone (Sorlie et al., 2005). Nordam, Torjuul, and Sorlie (2005) revealed the ethical challenges in male nurse care of older people. The nurses in this study indicated difficulties cooperating with nurses in other wards and frustration at having to use coercion or restraint while caring for patients with dementia. Nolan (2007) described the following experiences of nurses who cared for older people with dementia: structural inadequacies of acute care hospitals as a setting for dementia care, frustration due to limitations on care imposed by structural inadequacies, and complications from the continually changing needs of people with dementia. Furthermore, Borbasi, Jones, Lockwood, and Emden (2006) clarified the following factors that influence the management of patients with dementia: time pressure, overwork, lack of resources, and lack of knowledge/understanding of dementia among medical, nursing, and other health care professionals.

Issues regarding nursing care for patients with dementia in acute care hospitals in Japan may differ from those in other countries because the working environments of Japanese nurses differ from those in other countries. For example, nurse staffing levels are arranged according to supply in Japan but according to need in the USA and the UK. As a result, fewer nurses work at night than during the day in Japan (Yasukawa, 2005). In addition, especially in acute care hospitals, the number of years of nursing experience is very short in Japan (70% of nurses had <10 years of experience, and about half had <4 years of experience; Kanai-Pak, Aiken, Sloane, & Poghosyan, 2008). This length of nursing experience is shorter than that in Western countries such as the UK and Canada (Aiken et al., 2011). Therefore, nurses in Japan have limited opportunities to consult expert nurses. Moreover, many families of patients with dementia in Japan have had the experience of being asked to attend to their family member in hospital (Hattori, Takeda, & Sasaki, 2010). About 90% of nurses also report having asked a family member of a patient with dementia to attend to the patient in hospital (Kitai & Nakayama, 2012). In Japanese culture, the cooperation of the family is thought to be indispensable in the case of a family member with dementia being hospitalized.

Previous Japanese studies have revealed that nurses had difficulties accepting patients and their situations. The tensions associated with routine work with these patients, conflicts with their families, and treatments unique to dementia care were all challenging; the nurses feared the increased accident risk and spent more time administering care only to patients with dementia (Taniguchi, 2006; Yamamoto, Yoshinaga, & Ito, 2010). These Japanese studies targeted the setting, including recuperation and long-term health care facilities. However, nurses in acute care hospitals may face difficulties unique to their setting because patients with dementia receive priority treatment and various restrictions apply to their treatment.

Accordingly, the goal of this study was to explore the difficulties and issues faced by nurses in providing care to patients with dementia in acute care hospitals in Japan. Identifying the difficulties and issues involved in this type of care may provide a basis for discussion of improvement measures and future directions of care administration to patients with dementia.

## Definitions

In this study, an acute care hospital is defined as one that mainly provides general sickbeds (Health and Welfare Statics Association, 2007). The phrase “patients with dementia,” as used in this study, includes not only patients diagnosed with dementia but also those judged by the nurse participants to have dementia on the basis of their experience. In Japanese acute care hospitals, 15–20% of the inpatients have dementia, although most of them do not have a diagnosis of dementia (Hattori et al., 2010).

## Methods

### Setting

The study was conducted in the western region of Japan. Hospitals with more than 100 beds and those with both surgical and internal medicine departments were approached. Hospitals were selected on the basis of maximum variation sampling according to size. Hospital size was classified into three groups (100–299 beds, 300–499 beds, more than 500 beds), and three to four hospitals were selected from each group. As a result of maximum variation sampling, 10 hospitals were selected and asked to participate in the study. Six hospitals agreed to participate. The remaining four hospitals declined participation in the study because of the extremely small number of patients with dementia in the hospital or lack time to participate.

### Participants

Nurses in multiple surgical and internal medicine wards participated in this study. Nurses working in intensive care units, those with <3 years of nursing experience, those without experience in provision of care to patients with dementia in their currently assigned ward, and head nurses were excluded.

Criterion sampling was used. Directors of nursing departments in the participating facilities were aware of the exclusion criteria. These directors selected participants after consulting with the head nurses of each ward. They were asked to choose nurses with various levels of experience from multiple wards. Five to ten nurses were selected from each hospital, and the total number of nurses was 50. The head nurses then requested selected nurses in each hospital to participate in the study.

All participants received both an oral and a printed explanation of the study from a researcher (RF). All the nurses who asked to participate in the study agreed to the request and provided written consent to participate.

### Data collection

First, the participants were asked to complete a questionnaire regarding individual characteristics and wards. The questionnaire covered items regarding the participants themselves, such as number of years of experience in nursing, currently assigned ward, clinical departments for which they had worked in the past, age, and sex. Other questions regarding wards concerned the total number of admitted patients and the number of patients with dementia.

After completion of the questionnaire, focus group interviews (FGIs) were used to collect data in this study. FGI is a suitable exploratory method to identify the issues experienced by clinical nurses who care for patients with dementia at acute care hospitals (Kitzinger, 1995; Krueger & Casey, 2008; Morgan, 1996, 1997). It is unusual for clinical nurses to provide care for patients with dementia in acute care hospitals. The FGI method helped participants to recall their past experiences with patients with dementia through interaction with other nurses (Krueger & Casey, 2008; Morgan, 1996, 1997).

Data were collected from February to December 2008. FGIs were conducted in every hospital, and FGI sessions lasted 1.0–1.5 hr and were held in meeting or training rooms at each facility. The FGI environment in which the interviews were held was private. Only the participants and researchers were included in the sessions.

The FGIs were conducted as follows. One researcher (RF)—acting as moderator—provided topics of discussion and copies of the interview questions, which are shown in Table I. Discussion was refocused to the intended topic when the discussion digressed. The sessions began with the open question. Responses were recorded using an integrated circuit recorder and field notes. Field notes were written by a clerk. Key words associated with each participant remark or nonverbal response (e.g., facial expression, posture) and descriptions of the overall atmosphere were recorded for later use.

**Table I:** Interview questions.

What difficulties or issues did you experience when caring for patients with dementia who were receiving surgical or other internal medical treatments?
What measures did you take in previous difficult situations or incidents? Furthermore, why did the problematic situation occur?
Have you felt any ethical dilemmas while caring for patients with dementia? If yes, please explain.
Have you had any difficulties obtaining cooperation from a patient's family or any differences of opinion with them? If so, please explain. If yes, what measures did you take in these cases?
Does your hospital have any policy regarding admission of patients with dementia? If yes, please explain.

### Analytical methods

The qualitative synthesis method originally developed by J. Kawakita (KJ method) was adopted in this study (Kawakita, 1967, 1970; Yamaura, 2008). This method has been used in a wide range of fields, including industry, academia, and local and public administration, for more than 50 years. It has also been used recently in nursing to decode hypothetically random, unexplained phenomena and form them into a relevant, rational framework (Yamaura, 2008). This method was helpful in extracting issues in nursing care from participant data regarding their facilities.

Three processes were involved in the KJ method: code making, grouping, and chart making (Kawakita, 1967, 1970; Yamaura, 2008). Recordings from the integrated circuit recorder were transcribed. The data obtained were then unitized by focusing on issues experienced during care of patients with dementia and by breaking down sentences into the smallest possible units so as not to eliminate the participants’ thoughts. The unitized data were identified as codes. This process is called code making.

**Figure 1:**
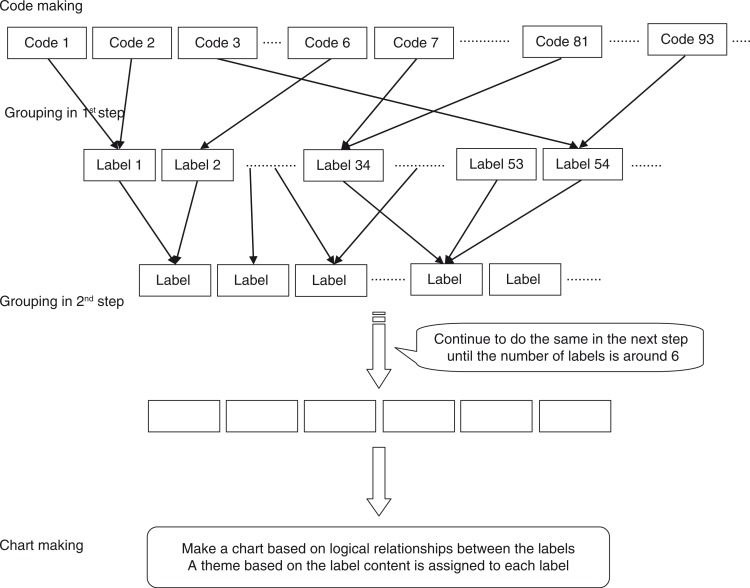
Analytical methods.

The codes were formed into groups based on similarities between the main themes under each code. After the initial grouping, a short summary was made to describe the essence of all codes belonging to each group. This short summary was used as the label to symbolize the group in the next step; this was the first step in the process of grouping. The same process was repeated with these labels (Figure 1). The grouping process was repeated until approximately six labels were formed, which were arranged spatially into a chart. The logical relationships between the labels in the final grouping were identified. The content of the labels in the final grouping were expressed in a short phrase, which became the descriptive theme assigned to the short summaries. This process is called chart making. The analyses described above were performed for each hospital. Thereafter, the results of these analyses were unified.

### Ensuring credibility and authenticity

During data collection, a researcher (RF) who was trained in interview procedures and had previous FGI experience acted as a moderator. The moderator summarized some of the participants’ remarks to confirm that the intentions of the remarks were clear. Prior to the analysis, this researcher took two training courses in the KJ method. The study was supervised by a faculty mentor (YS) who had full knowledge of the method. Nurses with experience in administering care to patients with dementia in acute care hospitals verified the logical relationships between the final labels.

### Ethical considerations

This study protocol was approved by the Ethics Committee, Division of Health Sciences, Faculty of Medicine, Osaka University (Ref. no. 57). The directors of the nursing departments and the participants in the cooperating facilities were informed of the study's purpose and methods verbally or in writing. Written informed consent was obtained from all participants, who understood that participation was voluntary, that they could leave the FGI at any time, that all information would be treated confidentially, and that the data would be stored securely and destroyed following completion of the report.

## Results

### Participants

The number of beds in the six studied hospitals ranged from 188 to 1021 (average: 417). The number of participant nurses from each facility ranged from 5 to 11 (total participants: 50; 49 female). The characteristics of the participants and their workplaces are shown in Table II. The average age of participants was 32.3 years (average years of experience: 9.8).

**Table II:** Demographic data of participants and focus groups.

Hospital	A	B	C		D		E	F	
							
Focus group	A	B	C1	C2	D1	D2	E	F	Total
Number of participants	6	6	6	5	6	6	5	10	50
Gender									
Female	6	6	6	5	6	6	4	10	49
Male	0	0	0	0	0	0	1	0	1
Average age (*SD*)	34.2 (14.4)	33.7 (6.2)	25.8 (2.0)	29.4 (7.2)	34.6 (5.5)	34.6 (3.1)	40.4 (8.7)	29.3 (4.5)	32.3 (7.9)
Age range	22–53	26–41	23–29	23–43	30–45	29–38	31–50	25–38	22–53
Average nursing experience (*SD*)	13.3 (12.4)	10.7 (5.8)	5.0 (1.5)	8.3 (7.0)	12.4 (5.9)	8.8 (2.9)	15.0 (5.5)	7.1 (4.9)	9.8 (6.8)
Range of nursing experience	3–30	5–20	3–7	3–20	8–24	5–13	9–24	3.9–18	3–30
Department									
Internal medicine department	1	0	6	0	3	4	1	2	17
Surgical department	0	0	0	1	2	2	2	1	8
Mixed ward with internal medicine and surgical patients	1	4	0	4	0	0	0	7	16
Others	4	2	0	0	1	0	2	0	9
Average number of inpatients per ward (*SD*)	55.7 (8.0)	41.8 (1.8)	45.9 (4.3)		36.3 (6.7)		29.0 (5.8)	46.9 (3.8)	42.8 (9.1)
Range of number of inpatients per ward	40–60	40–45	40–53		30–50		22.5–35	40–50	22.5–60
Average number of inpatients with dementia per ward (*SD*)	39.0 (17.5)	3.6 (1.6)	22.0 (13.6)		5.1 (3.7)		2.4 (1.2)	2.5 (1.4)	11.2 (14.6)
Range of number of inpatients with dementia per ward	10–55	1.5–5	10–40		1.5–10		1–4	0.5–5	0.5–55

### FGI outline

Eight focus groups of 6–10 participants each were formed. The characteristics of the focus groups are provided in Table II. The average time spent in FGI sessions was 64.1 min.

### Relationships between issues faced by nurses caring for patients with dementia in acute care hospitals

**Figure 2:**
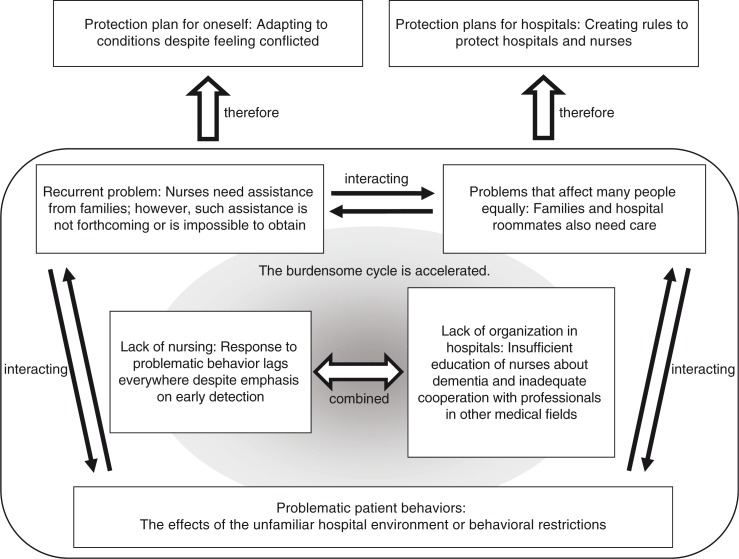
Schematic diagram of the issues faced by nurses caring for patients with dementia in acute care hospitals.

The data acquired from the FGI sessions were structured using seven themes, and the interrelationships between the themes are shown in Figure 2. On the basis of these relationships, we can draw conclusions with regard to the issues faced by nurses caring for patients with dementia in acute care hospitals. Problematic patient behavior affects many individuals, including the families and hospital roommates of patients with dementia. Therefore, families and hospital roommates may also require nursing care for fear, anxiety, and frustration related to the problematic behavior of patients with dementia. Families are, however, also regarded as assistants when patients with dementia are hospitalized in Japan, and they are regarded as essential to prevent the problematic behavior of the patient and to protect the patient's safety. In consequence, problems arise when the problematic behavior of the patient with dementia is repeated when the assistance of the family is impossible to obtain.

These interrelated problems indicate that a burdensome cycle is at work in acute care hospitals in which nurses are expected to care for patients with dementia. This cycle is exacerbated by two other problems identified in this study: lack of nursing experience/training regarding patients with dementia, and lack of organization/cooperation among professionals in various medical fields in acute care hospitals. Nurses reported that they adapt to the above-mentioned cycle by protecting themselves; thus, protection plans for hospitals must be implemented to avoid liability issues.

### Issues faced by nurses caring for patients with dementia in acute care hospitals

In this section, we outline the issues faced by nurses caring for patients with dementia in acute care hospitals identified in the study. Themes are provided, along with some codes as examples of the responses obtained during FGI.

#### Problematic patient behaviors

“The effects of the unfamiliar hospital environment or behavioral restrictions.”

Various environmental changes, anxiety when patients see no familiar faces at the hospital, painful treatments, and physical restrictions cause problematic behaviors such as falling and wandering. The codes indicate that patients with dementia feel a sense of security and calm down when spending time with their families.

If no family member attended to the patient during the day, the patient was likely to become angry and abusive. Thus, a patient's sense of security and behavior depends on whether or not a family member is present. (B hospital)

Patients with dementia had difficulty adhering to the restrictions of medical treatment and the hospital environment.

Many patients fall on the day of or within 2–3 days of admission to the hospital, because they often wander around an unfamiliar environment during that period. In addition, nurses might not be able to adhere to fall prevention protocols depending on the fall risk of the patient, because they cannot achieve an adequate understanding of the patient's fall risk. (E hospital)

#### Recurrent problem

“Nurses need assistance from families; however, such assistance is not forthcoming or is impossible to obtain.”

Many problems occur during medical treatment and hospitalization of patients with dementia, such as dangerous conduct, unhygienic behavior, and inability to abstain from food when required. These problems cannot be solved by nurses’ efforts alone, and cooperation from families is required.

Patients with dementia often have trouble understanding their circumstances, and as a result, they lash out irrationally. In such cases, nurses take measures to prevent dangerous behavior; however, they cannot fully prevent such behavior (i.e., measures are not always successful). In addition, especially in staff-shortage situations (e.g., during the night), nurses find it difficult to provide care even for patients without dementia. Therefore, patients with dementia can often receive adequate care in the acute care setting only with the cooperation of their families.

Even when I hid the intravenous infusion line in a bandage or a long-sleeved shirt so that it was not visible to the patient, the patient had already extracted the intravenous infusion line by himself/herself the next time I checked. (D hospital)

There are times when a nurse will request that a family member of a patient with dementia comes in, such as when a patient becomes angry and abusive at night, leaving the nurse unable to tend to other patients. (A hospital)

However, the nurses in this study reported difficulties obtaining cooperation from patients’ families. In addition, many patients with dementia are older adults; therefore, their family members are often also older, or they may have no families at all. In such cases, nurses face problems with obtaining informed consent regarding care and treatment.

Because patients with dementia cannot take care of a stoma by themselves, we have to ask the family to learn how to perform this task. Even if family members understand how to take care of a stoma after explanation, it is difficult for them to maintain a positive attitude, because taking care of a stoma entails disposal of feces. (D hospital)

Obtaining informed consent regarding care and treatment is impossible in the case of some patients. Many are older, and increasing numbers of patients have no family. Thus, the way in which informed consent regarding care and treatment will be acquired in the future is an issue. (A hospital)

#### Problems that affect many people equally

“Families and hospital roommates also need care.”

Families and hospital roommates require care in many situations.

Hospital roommates often feel uneasy or scared because of the behavior of patients with dementia. Thus, the nurses in this study felt that it was necessary to provide care for these roommates.

Because of problems related to patients with dementia, such as opening other patients’ curtains during the night, using other patients’ portable toilets, and opening other patients’ drawers without permission, patients sharing the same room become afraid. I realized that care for surrounding patients is also necessary. (F hospital)

Family members may become frustrated with and abusive toward patients with dementia, which can cause problematic behavior in the hospital. Their lack of understanding is a hindrance to the patient's medical treatment.

In digestive surgery, one patient used to remove his own intravenous infusion line every day. He removed his drain by himself and kept repeating the same actions. His family members must have been irritated, and although I do not allege abuse *per se*, the patient was hit when no one was watching. (F hospital)

Although the nurse kept beverages away from the bed of an older person who was in the hospital for heart failure, his symptoms did not improve. Later, we found that the wife had been giving the patient water at his request. (D hospital)

Sometimes, family members are also older adults with dementia. They may also cause trouble with other patients and often do not understand the concept of informed consent about care and treatment. As a result, treatment does not progress.

In one case in which the person attending to the patient was an older adult, forgetfulness was common. He would borrow money for the washing machine from other patients and forget to repay them or wash other patients’ laundry. This got him into trouble with other patients. (C hospital)

#### Lack of nursing

“Response to problematic behavior lags everywhere despite emphasis on early detection.”

Nurses understand that they must detect any signs of problematic behavior as early as possible, which can be achieved by close observation of regular behavior, noticing small differences in daily activities, and comprehensive analysis of examination results. However, prevention of incidents and detection of problematic behavior tends to lag behind because of the difficulties associated with understanding the symptoms of patients with dementia.

In most cases, nurses reported that they noticed the signs and causes of incidents only after the fact. Patients tripped over containers and furniture, fell, made a scene, or showed sudden change in their emotional state. All such behaviors can be avoided through advanced planning and countermeasures. Each patient with dementia has a unique way of expressing symptoms. Observation provides the opportunity to notice minute changes.

One patient did not eat his/her meals at all and came to dislike even seeing food. I thought that this was because of the dementia; however, the actual reason was a cancer metastasis. (C hospital)

The following codes indicated that nurses interviewed in this study had difficulty preventing incidents because they often did not understand their causes.

Because we collect patient information during patient care, a patient may fall before it is judged whether he/she is prone to falling. Although we sometimes think that we should have been more careful, it is difficult to anticipate and deal with such situations in advance. (E hospital)

#### Lack of organization in hospitals

“Insufficient education of nurses about dementia and inadequate cooperation with professionals in other medical fields.”

Nurses have no opportunities to obtain knowledge and advice regarding dementia. Nurses in this study did learn from their own experiences and their observation of how senior nurses handled patients with dementia. Although difficult cases were discussed during conferences, issues sometimes could not be resolved, or the time to discuss the issues during conferences could not be secured. Thus, nurses were often left to solve issues related to dementia care themselves.

Nurses do not have opportunities to acquire specific knowledge or advice about dementia. Therefore, they deal with problems as they occur. (B hospital)

When I was a junior nurse, I did not know how to care for patients with dementia. At that time, I learned how to care for patients with dementia by observing senior nurses. (D hospital)

The data indicated that acute care hospitals had organizational problems related to infrastructure and insufficient cooperation with professionals in other fields.

Because the nurse call button is not connected to a personal walkie-talkie system, it cannot be heard when nurses are administering care to a patient in a room far from the nurses’ station. (C hospital)

Doctors’ understanding of patients with dementia is limited; therefore, even if a nurse asks for a psychiatric consultation, the doctor might say that a consultation is not necessary for that level of dementia and refuse to conduct one. (F hospital)

Cooperation between psychiatrists, assisting nurses, and hospital security staff is insufficient. In an acute care hospital setting, although the number of nurses available appears to be sufficient, sufficient care cannot be provided to patients with dementia because of the multiple other demands on their time. The organization and structure of hospital systems are inadequate to meet the needs of patients with dementia.

#### Protection plans for hospitals

“Creating rules to protect hospitals and nurses.”

Measures must be taken to respond pre-emptively to issues that could lead to liability problems for the hospital. The data gathered in FGIs imply that nurses meet the demands of hospital administration through the following measures: they may only admit patients who have somewhere to go after discharge, who have agreed to leave 2 weeks after admission, or whose family members can take care of them after discharge; these measures may be necessary to maintain their nursing positions.

Because we were informed of low bed turnover rates, we have not been admitting patients unless we know that they will be transferred, and we ensure with indications on the medical chart that the patient will be transferred to another hospital after 2 weeks when consent is obtained from the patient's family. (F hospital)

Although nurses take all possible measures to prevent falls and extraction of intravenous infusion lines, accidents still occur. Indicators of their efforts include placing sensor mats before accidents occur and taking patients with dementia on rounds; however, nurses may still be blamed by the patients’ families for these accidents.

When a patient falls and problems ensue, the family may ask the nurse, “Why did you not prevent the fall?” or “Why did the patient fall down?” The use of a sensor mat is an indication to families that we take many precautions. (F hospital)

#### Protection plan for oneself

“Adapting to conditions despite feeling conflicted.”

In terms of care administration to patients with dementia in acute care hospitals, nurses feel a gap between the ideal situation and the reality of a negative atmosphere. Acute care hospitals aim to treat acute diseases. For various reasons, nurses feel extra pressure when administering care to patients with dementia in such hospitals, as indicated by the following code.

Nurses attempt to ensure that patients with dementia do not fall, for example, by bringing them to the nurse's station or taking them on rounds. Nevertheless, nurses are blamed by doctors or other medical workers if the patients end up falling anyway. However, in an acute care hospital, nurses may suddenly need to attend to other patients because of rapid changes in their condition. Therefore, working in such a setting is very difficult. (D hospital)

Nurses question practices in which medical treatments and life-supporting measures are prioritized, even when they lead to patients being restrained or restricted physically. For example, very old patients (i.e., those aged >90 years) may be taken to rehabilitation or be given treatments against their will; the same may occur in those with poor prognoses on the basis of their families’ request.

Reinsertion of central venous catheters is a physical burden on the patient. Thus, the patients’ movement must be restricted to prevent them from tampering with or removing necessary equipment. This is accomplished using things such as mittens, overalls, and physical restraints. (C hospital)

In the case of a terminally ill patient in the internal medicine department, surgery was no longer possible, and the only option was chemotherapy. The patient did not understand his/her situation, but his/her family did and consented to chemotherapy. Although we administered chemotherapy to extend the patient's life by approximately 1–2 months, this medical treatment is hard on the patient. I wonder if this is really necessary. Many people feel that it may be better for the patient to go home and spend time eating what he/she likes and doing what he/she pleases. (F hospital)

Nurses also recognize the dilemma of their lack of time to communicate with patients, although such communication would help to maintain the patients’ cognitive function. In other cases, nurses adjust patient schedules so that they can perform their duties more smoothly, because acute care hospitals cater for a large number of patients. Moreover, nurses adapt to such circumstances.

There is always a gap between the ideal situation and reality; I know I need to stand up for the patient's side, but for work to proceed smoothly, I sometimes get the patient to adjust to me. However, I am becoming accustomed to these situations. (A hospital)

## Discussion

### Relationships between issues faced by nurses caring for patients with dementia in acute care hospitals

In this study, we identified a cycle that occurs in the care of patients with dementia in acute care hospitals. Nurses are required to deal with patients’ problematic behavior. They take measures such as hiding intravenous infusion lines and placing sensor mats to prevent problematic behavior. However, these measures may make the environment less familiar to patients with dementia, resulting in an increase in problematic behavior. Studies have found that environmental stimulation exaggerates the stress response in patients with dementia (Cunningham & Archibald, 2006; Fetzer, 1999; King & Watt, 1995; Martin & Haynes, 2000). The present findings support this idea.

Borbasi et al. (2006) recognized that the family is important in the acute care setting and reported that although family members are generally considered as beneficial assets to patients and staff, they often require substantial support themselves. The results of this study were similar to those of previous studies. In this study, nurses could not resolve certain issues alone when patients with dementia were recovering from medical treatment; thus, the nurses turned to the patients’ families for help. At the same time, they understood the necessity to care for the family. Their input during the FGI sessions indicated a mental struggle between asking for help from families in resolving difficult problems and the need to support the same family members.

A previous study reported that nurses were frustrated by the lack of time to spend with and speak with patients (Sorlie et al., 2005). This issue is an ethical challenge in acute nursing care (Sorlie et al., 2005). Research on nursing home staff has found that nurses’ frustration, which is related to a lack of time, stems from the wide variety of diagnoses and reasons for hospitalization of the patients (Jakobsen & Sorlie, 2010). The nursing home study reported that one nurse said that they “had several patients with dementia for assessment in the short-stay unit. This takes time, and the disquiet they create leads to frustration among the staff” (Jakobsen & Sorlie, 2010, p. 295). Participants in our study might also have been in similar situations, needing more time to communicate adequately with patients with dementia. Simultaneously administering care to patients with and without dementia is extremely challenging. Our study showed that nurses feel conflicted because of their lack of available time to provide nursing care to patients with dementia and their families.

The cycle identified in the current study was exacerbated by the lack of nursing training/experience regarding patients with dementia and organization/cooperation among medical professionals in different disciplines in acute care hospitals. The data indicated that nurses realized that early detection was important; however, they had difficulties understanding symptoms, resulting in delays in the detection of incidents. They recognized the need for prevention of incidents in the care of patients with dementia; however, they felt that they were in danger of overlooking signs because of their lack of training and experience. Previous studies have identified overwork, lack of resources, and lack of knowledge and understanding of dementia among staff as difficulties in nursing provision for patients with dementia in acute care settings (Borbasi et al., 2006; Eriksson & Saveman, 2002; Nolan, 2007; Nordam et al., 2005). Lack of communication between nurses and other health care professionals made nursing care for patients with dementia at acute care hospitals more difficult (Sorlie et al., 2005). In this study, we suggest that these deficiencies in hospital systems exacerbated the cycle.

As stated in the previous paragraph, nurses reported facing various problems and difficulties caring for patients with dementia; these problems and difficulties interacted and resulted in a cycle. Despite this situation, nurses were eager to adapt to their present conditions. However, they felt pressure because of a lack of time and an inability to respect the patients’ wishes, as also reported in previous studies (Borbasi et al., 2006; Eriksson & Saveman, 2002; Nolan, 2007; Taniguchi, 2006; Yamamoto et al., 2010). Although nurses accepted their mission to follow their designated routines in their present conditions, they questioned the situation.

The results of this study clarified that nurses attempt to protect themselves and their hospitals under the organizational limitations characteristic of acute care hospitals. One previous study reported that securement of additional community services for people with dementia was long overdue (Borbasi et al., 2006). In another study, participants described “a constant demand from the top of the organization [to] decreas[e] … care time” (Eriksson & Saveman, 2002, p. 82). These studies indicate that the problems identified in our study are characteristic of the problems of nursing care for patients with dementia in acute care hospitals. Thus, nurses reported taking actions to protect themselves because of organizational pressure.

### Unique issues in the nursing of patients with dementia in acute care hospitals

The results of this study differed from those of previous studies conducted in long-term care facilities or nursing homes (Brodaty, Draper, & Low, 2003; Enes & de Vries, 2004; Jakobsen & Sorlie, 2010; Kada, Nygaard, Mukesh, & Geitung, 2009; Matsuda, Nagahata, Ueno, & Gora, 2006). The need to care for patients’ families and cooperate with them had not been reported in previous studies of long-term care facilities or nursing homes for the following reasons. Long-term care facilities or nursing homes provide care to people who require full-time help and cannot receive support from family and friends (Ministry of Health, Labour and Welfare, 2010; National Institutes of Health, 2014). In addition, long-term care facilities or nursing homes in Japan aim to relieve caregivers’ physical and mental load (Ministry of Health, Labour and Welfare, 2010). Therefore, nurses working in long-term care facilities understand that they cannot gain assistance from families. These nurses also believe that by caring for patients with dementia in these facilities that they are also caring for the patients’ families.

Many patients with dementia are admitted to long-term care facilities (Awata & Watari, 2007; Lithgow et al., 2011; Miura et al., 2005; Sandberg et al., 1998; Yamasaki & Kodama, 1995). For example, Miura et al. (2005) found that 65.2% of inpatients with dementia in Japan were in psychiatric wards, and 24.9% were in recuperation facilities. Furthermore, Sandberg et al. (1998) found that in Sweden, 18.9% of inpatients with dementia were in emergency hospitals, and 66.3% were in nursing homes. Thus, nurses working in these facilities may not feel the need to ask for assistance from families, unlike nurses working in acute care hospitals, because they are already familiar with care administration to patients with dementia. The above indicates that nurses’ need for cooperation with patients’ families regarding patient care and the need to care for the families themselves are characteristic issues in caring for patients with dementia in acute care hospitals.

### Suggestions for nursing practice

The results of this study suggest that the nurses interviewed lacked both observational methods with which to understand the symptoms of patients with dementia who could not communicate, and suitable measures to deal with problematic behavior. Previous studies in nursing homes and long-term care facilities have described the effectiveness of staff training programs on problematic behavior among people with dementia (Gould & Reed, 2009; McCabe, Davison, & George, 2007). By use of these training programs, acute care hospitals could provide an education system to train nurses in observation and coping methods comparable with those practiced in long-term care facilities.

In this study, nurses described being placed in situations in which they felt compelled to call on patients’ families to aid in the care of patients with dementia. Labor shortages and the misconception that care administration to patients is a job only for nurses contributed to this problem. Thus, managers in acute care hospitals must coordinate the efforts of different staff members so that nurses do not feel the need to request aid from patients’ families, and they must create an environment in which all staff within the organization understand that dementia is a disease. This study's participants stated that if nurses, other medical staff, and all staff in occupational medicine could work together, patients with dementia would benefit from better care. Early detection of problems and prevention of problematic behavior would be facilitated. Nurses would also be able to find more time to care for patients’ families and roommates.

Next, we make a recommendation to nurses in acute care hospitals in relation to falls prevention. Many participants in this study have experienced difficulty preventing patients with dementia from falling. Participants take all possible measures to prevent falls, such as observing patients with dementia and using sensor mats. Practice guidelines for fall prevention recommend that the risk of falls be assessed upon the occurrence of a fall, upon transfer to another unit, when a significant situation is likely to increase fall prediction factors, and on admission (Gray-Miceli, 2008; Health Care Association of New Jersey, 2009). In Japan, the manual for fall prevention created by the National Hospital Organization provides an assessment sheet for falls, which provides the same recommendation as the practice guidelines about the timing of risk assessment (National Hospital Organization, 2010). Therefore, we recommend that nurses assess the risk of falls at appropriate times and execute plans according to risk assessment results.

### Limitations

This study has some limitations. First, hospitals were selected by maximum variation sampling. However, most larger hospitals (i.e., those with more than 600 beds) refused to participate in the research. Thus, this study lacks data from larger hospitals and, therefore, the research findings may not fully reflect all opinions. However, the percentage of hospitals with more than 600 beds in Japan was only 3.0% in 2010 (Ministry of Health, Labour and Welfare, 2011b). Therefore, the research findings reflect the situation of care administration to patients with dementia in most hospitals.

Second, the nurse recruitment process in this study depended on the directors of nursing departments and head nurses. Nevertheless, participation in the study was voluntary, and nurses were allowed to withdraw from the study at any time. However, nurses might have been influenced to participate in the study by their directors and head nurses.

Third, data collected in this study were based on the recollections of nurses. Therefore, some data may be affected by recall bias. However, the nurses looked back on their past nursing experience by group dynamics and helped each other to remember their methods of care administration to patients. In addition, nurses may have been able to recall accurately the history of care administration to patients with dementia, as they could get this information from FGIs (Coughlin, 1990). Therefore, the influence of recall bias on the data may be minimal. However, some nurses might have been affected by the opinions of other nurses.

Fourth, the ratio of male to female nurses in this study was unbalanced (1 male to 49 female nurses). The proportion of male nurses in Japan was 5.6% in 2010 (Ministry of Health, Labour and Welfare, 2011a); therefore, the proportion of male nurses in this study was representative. However, the results may not adequately represent the opinions of male nurses.

Finally, generalization of these research findings may be difficult because this is an exploratory study. Although similar results were obtained from each hospital targeted in this study, the priority and importance of the problems discussed here may differ between hospitals. A quantitative study will be conducted to consider the differences in these problems in relation to hospital scale, specialty, and location. Such differences between hospitals must be considered in any plan for care administration to patients with dementia in the acute care setting.

## Conclusion

This study clarified two main problems experienced by nurses caring for patients with dementia in acute care hospitals: that various problems and difficulties interacted in a burdensome cycle and that nurses felt the need to adapt to their present conditions despite feeling conflicted. The necessity to care for patients’ families and obtain the families’ cooperation may be a general characteristic of care administration to patients in acute care hospitals.
